# Representation in participatory health care decision‐making: Reflections on an Application‐Oriented Model

**DOI:** 10.1111/hex.13483

**Published:** 2022-03-27

**Authors:** Tim Holetzek, Christine Holmberg

**Affiliations:** ^1^ Institute of Social Medicine and Epidemiology Brandenburg Medical School Theodor Fontane Brandenburg an der Havel Germany; ^2^ Faculty of Health Sciences Brandenburg Brandenburg Medical School Theodor Fontane Potsdam Germany

**Keywords:** health care decision‐making, legitimacy, participation, representation, responsiveness

## Abstract

**Context:**

The involvement of lay people in health care decision‐making processes is now the norm in many countries. However, one important aspect of participation has not received sufficient attention in the past and remains underexplored: representation.

**Objective:**

This paper explores the question of how public participation efforts in collective health care decision‐making processes can attempt to aim for legitimate representation so that those individuals or groups not present can be taken into account in the decisions affecting them. This paper argues that to make decisions that effectively address those affected, representation needs to be seen as a relevant part of any participatory setting. To support this argument, the paper outlines the concepts of participation and representation and transfers them to health care contexts.

**Results:**

A conceptual reflection on responsiveness and the characteristics of representative actors in representative‐participatory settings is introduced, which could provide actors planning to conduct participatory health care projects with tools to reflect on the merits and possible flaws of participatory constellations.

**Patient or Public Contribution:**

The paper contributes to improving public participation in health care decision‐making.

## 1 INTRODUCTION

The increasing presence of and demand for ‘patient participation’, ‘patient engagement’, ‘patient orientation’ and ‘patient involvement’ can be seen in many aspects of health care today, including national law‐making,^1^ health research^2^, ^3^ and actual doctor–patient interactions.^4^ However, we assume that participation is often at risk of being merely a token, and in this way, the positive effect or potential that comes with involving relevant actors is minimized. In this article, we therefore develop a concept of participation in health care settings that considers the participants' ability to represent as an essential condition for successful participation.

The trend towards participation can be traced back to social movements evolving in the 1960s, including those by women and students, or by those not only in the name of the environment but also in health care. The antipsychiatry movement, for instance, was at the forefront of demanding that lay people have a voice in decision‐making and be taken seriously as autonomous and political subjects rather than being stigmatized and confined to psychiatric institutions.^5^, ^6^ The common thread in these movements was the call for equal rights and to have a voice in decision‐making at all levels of governance, which was formerly seen as lobby‐driven and intransparent.^7^ Power relations between institutions, such as state administrations, but also actors like psychiatric hospitals on the one side, and citizens on the other, became increasingly contested and perceived as asymmetrical,^8^ which effectively led to what some refer to as a ‘crisis of democracy’.^9^ The crisis in effect was seen as one where represented individuals no longer saw themselves represented by those in decision‐making power. As a result, participation in health care became an increasingly discussed and addressed issue. The World Health Organization's Alma‐Ata declaration, for example, has taken up this demand towards health care systems, declaring that ‘people have the right and duty to participate individually and collectively in the planning and implementation of their health care’.^10^ ^,^^p.1^ This demand has since been reinforced on several occasions, and nowadays systems of participation in health care have been legally fixed in various countries around the globe.^11^ Thus, public participation—in health care often referred to as lay or patient participation—has been recognized as crucial for responsive health care systems.

When it comes to public involvement, the terms ‘participation’, ‘involvement’ and ‘engagement’ are often used interchangeably.^12^ In accordance with Castro et al.,^13^ ^,^^p.1229^ we understand public participation in health care as ‘the contribution of patients or their representing organizations in shaping health and social care services by means of active involvement in a range of activities at the individual, organizational and policy level that combine experiential and professional knowledge’. This contribution is especially relevant to decision‐making processes, which are usually defined as processes or sequences of activities that are initiated upon the recognition of an underlying problem or condition and usually result in a decision to act or not to act to respond to such problem or condition.^14^ Such processes can be found at all levels of a health system and can be small or large in scope. For the purpose of this article, we thus make use of ‘participation’ as a reference term and understand it as the involvement of lay people in decision‐making processes. By involving lay people in such processes, their voices are actively considered. It is thereby assumed that democratic processes of society are improved, as involving more directly those who experience the effects of the decisions to be made increases the democratic character of public decision‐making.^12^, ^15^, ^16^ This, in turn, raises the acceptance of public decisions among citizens and is assumed to improve the responsiveness of health care systems to citizens' needs and may thereby ensure that decisions have greater legitimacy.^17^

Evidence on the effects of involving lay participation in such processes is, however, scarce^18^ and a range of problems have been identified with the concept.^12^ In this paper, we argue that the key to a more democracy‐enhancing way of conducting participation in health care is reflecting on representation—a concept that has so far received only marginal consideration in this context. While being a pivotal element to the democratization of health care, the question of how legitimate representation comes into being in participatory health settings is only marginally taken into account. Because it is assumed that lay participation in health care, policy‐making, and research improves democratic processes as well as the responsiveness of the health care system to its citizens' needs, we argue that representation as a key principle of democracy needs more scrutiny. We therefore first outline the margins of legitimate representation, before the insights on participation and representation are merged and transferred to health care. In a final step, a conceptual reflection on legitimate representation is introduced, which we suggest can help to ensure the potential of participatory initiatives in health care decision‐making settings and to identify examples of insufficient representation. In this way, especially those institutions that plan to involve patients or other groups of people in health care decision‐making processes may gain insights into how this can be achieved in such a way that the resulting decisions also meet the interests of those who do not participate, thereby increasing the decisions' acceptability.

## 2 THE LOGIC OF REPRESENTATION

Representation is a form of social interaction in which different types of actors are brought into relation to each other.^19^ Each such social interaction includes at least two types of actors: those being represented (also called the constituency) and those representing them. Representatives are actors that speak, advocate, symbolize and act on behalf of others. Their task is thereby to ensure that those represented have a stake in the action. Representatives thus have to ensure that the actions ‘are being performed not just on behalf of the represented but also in the name of the represented’.^20^ ^,^^p.96^ By speaking and acting on behalf of others who cannot be present themselves, representatives allow these others to be present by making certain characteristics visible. Representation can relate to many things, including preferences, interests, identities and values.^21^ In this way, representation circumvents the impossibility of involving all people affected by potential decisions.^22^

Probably the most prominent approach to this topic was made by Pitkin,^23^ ^,^^p.144^ who highlighted the often‐used etymological meaning of representation as ‘making present again something that is not literally present’. She further defined a representative as ‘someone who has been authorized to act’.^23^ ^,^^p.38^ According to this approach, the represented individuals' will could be seen as evident and objectively tangible by the representative. Pitkin's understanding of representation was, however, rather static and normative and limited to the political field. Later approaches, like those of Rehfeld or Saward, expanded the approach to more constructivist spheres, according to which representation could be perceived in terms of dynamic processes between those being represented and their representatives.

Saward conceptualized representation ‘not as a fact that results from election, but rather as a process of making claims in electoral but also many other contexts’.^24^ ^,^^p.1003^ He did so by developing a theory of the representative claim, according to which actors are in an ongoing competition to claim the right to represent certain groups of individuals.^25^, ^26^ These claims can be made not only by elected but also nonelected actors in either implicit or explicit ways. The dynamic of representation is then characterized by some sort of relationship between those claiming to be representative of someone and those being represented. Accordingly, representatives and their constituency need to be in an interactive relationship that, on the one hand, allows the representatives to make visible their claim to represent and, on the other, gives the proclaimed constituency the opportunity to evaluate such claim.^19^, ^26^ Furthermore, the will of the represented individuals is interpreted and constructed by the representatives. The iterative process of interpretation and construction is guided by the dynamic interaction of represented and representing individuals. Hence, the relationship between the actors involved is regularly treated as an indication of the legitimacy of the actions performed by the representatives. This relationship is generally termed *responsiveness* and is conceptualized as a mechanism of accountability and authorization.^21^, ^27^ While authorization refers to how ‘representatives are selected or directed’ by those they represent,^28^ ^,^^p.360^ accountability refers to how representatives explain and justify their actions towards those they represent^29^ (as cited by van de Bovenkamp and Vollaard).^30^ ^,^^p.102^ Besides elections, various other manifestations of such mechanisms are thinkable and in place, for instance (but not limited to) starting or terminating membership of an organization, organizational meetings in which the representatives' actions need to be approved by the members, publishing publicly available annual reports.^21^, ^27^ The responsiveness criterion is not only exclusively applicable to elected representatives but also to a wide range of self‐authorized actors, which can be considered democratic if they, too, demonstrate responsiveness with the groups they claim to represent by means of the mechanisms mentioned above.^25^, ^27^

Although the concept of responsiveness is conceptually quite well developed and follows a certain tradition in political theory (for an overview, see Rehfeld),^19^ it remains empirically underexplored.^31^ Van de Bovenkamp and Vollaard^30^ have transferred the topic to health care settings. The authors explore the mechanisms of authorization and accountability of those actors claiming to represent patients in the Dutch debate on centralization of care. They conclude that in this context, a wide variety of actors claims to represent patient collectives and that responsiveness is upheld in various ways. Fischer and van den Bovenkamp go on to show the wide range of mechanisms used by patient organizations (POs) to transform representative aspirations into democratic representation. The authors conclude that especially informal mechanisms play an important role in this process. They show the importance of informal mechanisms that play an essential role in the process of representation.^32^ We build on this study and expand it with regard to the elicitation of concrete consequences of dynamics that characterize representation. We would argue that a purely descriptive list of existing mechanisms remains largely uninformative unless they are linked to real‐world consequences. These can be empirically elaborated in a practical way by means of decision‐making processes in which the dynamics consequently manifest themselves in outputs, that is, decisions. In this way, we can focus on the influence of certain dynamics on decisions, which affect those who are represented. Therefore, we advocate focusing on concrete decision‐making settings.

## 3 PARTICIPATION AND REPRESENTATION IN HEALTH CARE SETTINGS

In the course of the past decades, several frameworks have been developed to enable lay people to participate, which address the activation of participants^2^ as well as the levels of integration in decision‐making.^3^, ^33^ Especially those frameworks focusing on the transfer of power have been influential.^33^, ^34^, ^35^ A prominent example is surely Arnstein's eight‐level typology of citizen participation, which has been taken up by many other scholars working in patient involvement.^33^, ^36^ With the help of this typology, the degree of power that participants receive or hold in participation processes. According to Arnstein, the lower levels of this model can be equated to ‘nonparticipation’ or ‘tokenism’ and the highest levels as ‘degrees of citizen power’.^35^ For the health care sector, Cahill has refined the concept for participation in health care to three levels: ‘patient partnership’, ‘patient participation’ and ‘patient involvement/collaboration’. Here, the partnership is the strongest level indicating power, while collaboration is the weakest type of power‐sharing with patients.^36^, ^37^ Similarly, Charles and DeMaio^33^ suggest a hierarchical three‐level typology for participation in health care settings: ‘consultation’, ‘partnership’ and ‘lay control’.^33^ According to the authors, consultation represents the lowest form of actual lay participation; although individuals can express their views on potential decisions, there is no guarantee that these views will be taken into account.^31^ Partnership allows for shared decision‐making competencies between the actors taking part in a given decision‐making process. Lay control, by contrast, provides the opportunity for autonomous decision‐making authority on the part of public participants.^33^, ^38^

In health care settings, participation is used not only in various areas, such as service development, planning, the delivery and evaluation of care, the education and training of health care providers, health technology assessment and research,^39^, ^40^, ^41^ but also in political decision‐making.^33^, ^40^, ^42^ The people who are involved in decision‐making can vary greatly, even though most of the time they consist of lay people and professionals (sometimes referred to as public and nonpublic members). Nonpublic members or professionals may be, for example, health care providers, members of the ministry of health or the public health system, as well as health researchers.^33^, ^40^ Public or lay participants can be patients, service users, health consumers, citizens, taxpayers and the like.^12^

Systems of lay participation in health care planning and research are more or less strongly implemented in most high‐income countries. In Germany, for example, lay participation in health care planning has been continually expanded over the past decades. The main decision‐making body on health care planning in Germany is the Federal Joint Committee (FJC; German: *Gemeinsamer Bundesausschuss*). Its main task is to define the detailed content of health care and to decide which services are to be included in the benefits catalogue of the German statutory health insurance. To perform this task, the FJC passes guidelines that have the status of sublegal norms and are legally binding for all actors within the statutory health insurance. This body of decision‐making includes members appointed by the Central Federal Association of Health Insurance Funds, the German Hospital Federation, the National Association of Statutory Health Insurance Physicians and the National Association of Statutory Health Insurance Dentists. In addition, four POs are allowed to appoint representatives who have the right to codetermination and make motions. Taking a closer look, it becomes clear that the selection of appropriate patient representatives is an administratively complex and demanding process. According to article 140f of the German Social Code, Volume V, the four relevant patient and self‐help organizations are responsible for proposing and mutually appointing patient representatives in the context of the Coordinating Committee of the FJC.^43^ The appointment criteria for patient representatives focus on technical and specialist competencies. Among others, half of the representatives to be appointed should themselves be affected persons.^44^ However, as new representatives are sought constantly and in high numbers for the numerous committees of the FJC, the necessary selection procedures may be pragmatically oriented and basically include only the legally prescribed criteria for the selection of representatives. Thus, they most likely do not weigh the relationship between the potential representative and the represented as a primary selection criterion. However, since the selection processes of the relevant POs are not transparently documented in detail, a dynamic relationship with the people they represent cannot be ruled out in principle. In our view, strengthening POs' internal legitimacy, which can be measured on the basis of a dynamic relationship between representatives and those represented, can be the first step toward lending legitimacy to the selection of patient representatives by POs. To do so, more transparency is needed to illuminate the relationship between patient representatives and those they claim to represent.

POs are probably the most influential representative actors in health care decision‐making and have been the focus of much scholarly attention in recent years.^45^, ^46^, ^47^ Individuals who are represented through POs can signal their approval by ‘signing petitions, attending rallies, joining the representative's social media groups or donating money’ and can in turn hold ‘representatives accountable for their decisions by withholding or withdrawing these forms of support or by publicly challenging the representative’.^48^ There are, nevertheless, many problems to be faced in this regard. An often mentioned aspect of POs is that they are increasingly professionalizing, meaning that patient representatives are highly skilled and educated people and sometimes not even patients themselves. As van de Bovenkamp et al.^49^ conclude, while professionalization is important for efficiently incorporating the views of the represented, it tends to keep representatives and the represented at a distance. The authors emphasize that by installing professionals as representatives, the experiential knowledge of patients is at risk of disappearing from the decision‐making process, which could ultimately lead to a negative impact on the democratic quality of representation and consequently risk producing a misrepresentation of the interests of individuals with different diseases or varying degrees of affectedness in the decision‐making process.^49^ Furthermore, when focusing on the responsiveness of POs, Baggott et al.^50^ ^,^^p.299^ distinguish between two forms of accountability, namely formal mechanisms like elections and annual general meetings, and less formal mechanisms ‘through consultative and participatory processes’. They argue that although most organizations hold elections, downward accountability is far more significant, such as through informal networks, interactions through newsletters, conferences and surveys and other actions.^50^ However, those being represented generally show only little interest in these accountability efforts and thus the representatives are held to account only to a minimal degree.^30^ POs thus present numerous problems when it comes to their relationship with those they represent, but to take advantage of the democratic quality of their representation, active and pragmatic reflection seems to be an essential first step. The same is likely true for most other participatory settings in health care. In this way, ‘bogus representation’,^51^ ‘token representation’^52^ or ‘not meaningful representation’^53^ can be limited.

## 4 AN APPLICATION‐ORIENTED MODEL OF REPRESENTATION

Considering the issues at stake, we now suggest aspects of responsiveness and characteristics of representative actors that may function as an evaluation framework or could help to structure participatory settings.

### 4.1 Responsiveness

When it comes to facilitating an interactive relationship between representatives and those they claim to represent, there are two possible ways to do so. Firstly, respective mechanisms of authorization and accountability may be created or altered (this idea is not new, see Parker),^54^ which may translate into a higher degree of interaction between the actors involved. To exploit their full potential, these mechanisms must be created to preserve not only occasional but ongoing interaction between representatives and the represented. A second way to facilitate responsiveness is to establish a beneficial relationship between a representative and their proclaimed constituency from the very beginning. We think that in health care settings it may be advantageous to look for descriptive forms of representation, according to which representatives should be a reliable sample of the represented ‘by being sufficiently like them’.^23^ ^,^^p.80^ A representative's suitability can hence be considered a priori on the basis of certain characteristics. While in the literature, descriptive representation is often associated with shared external characteristics like age, ethnicity, education and income, it can also be applied to the shared experience or history of a group.^55^ While it has been discussed intensively since the 1990s in the realm of civil society, the concept is increasingly being transferred to health care but is still in need of theoretical and empirical explication with regard to what consequences it entails for those being represented. It is often argued that when representatives share an experience with their proclaimed constituency, the two sides will be able ‘to read one another's signals relatively easily and engage in relatively accurate forms of shorthand communication’.^55^ ^,^^p.641^ Therefore, a descriptive representation based on shared experience facilitates communication between representatives and their proclaimed constituency, thus enhancing innovative thinking, underlining the ability to rule by the group and increasing the legitimacy of the representative and the decisions made.^55^, ^56^, ^57^, ^58^ In this way, descriptive representation can be understood as experiential representation and can become ‘a matter of common experience in the situation of the group represented’.^59^ ^,^^p.37^

To this end, a number of aspects must be taken into account, especially in the health care sector. Patients do not have common values or an identity that would distinguish them from other social groups. The experiences they have are based on experiences of illness, care and other aspects. These experiences do not represent a fixed quantity, but are always in flux and, moreover, can vary greatly, which is why they are not able to characterize groups of individuals appropriately. Therefore, the goal of descriptive representation cannot be to identify representatives who exhibit exactly the experiences or other criteria that a patient collective can exhibit. Rather, descriptive representation can help to establish a relationship of trust between representatives and their proclaimed constituency,^55^ which is also reflected in the fact that the represented see an opportunity for their own experiences and desires to inform decisions if their representative has had experiences similar to theirs.^58^

Mechanisms of authorization and accountability and the descriptive character of representatives are interconnected. While the former is indispensable for legitimate representation, descriptive representation can have a protective effect if such mechanisms are only insufficiently in place. Descriptive representation may therefore facilitate successful representation without necessarily being a prerequisite of it. Similarly, Dovi^60^ ^,^^p.730f.^ argues that ‘descriptive representation must entail mutual relationships and maybe skills of the representatives, and not just shared characteristics, so that the dynamic process of genesis of interests is considered’ (see also Arnesen and Peters).^56^ Together, mechanisms of authorization and accountability and descriptive representation may combine to enable a sufficient degree of responsiveness, which, in turn, could promote responsiveness across the health system and could effectively consider the needs of those affected by health‐related decisions.^61^, ^62^ Following on from Dovi,^60^ however, there are other skills and basic requirements to be considered that influence the outcome of participation and representation. These refer to the organizers of such settings and participants alike. However, the question of in what ways an actor is descriptively representative is arguably never easy to answer.

### 4.2 Characteristics of representative actors

In many cases, a participatory setting is organized by an actor who may or may not take part in the actual process of participation. These organizers shape the *who* and *how* of representation and also define the criteria used to judge whether or not the actions performed are representative. Furthermore, they may define and establish the mechanisms of authorization and accountability. Accordingly, they have immense power over the act of bringing representation into being.^12^ Especially those actors in charge of designing a particular decision‐making setting may anticipate the potential obstacles that come with power and representation. To do so, certain questions may be addressed beforehand: Does every participant in the decision‐making process have the same weight, or do professionals have a stronger voice? Are public participants allowed to vote on decisions? If yes, how are the final decisions made—unanimously, or by a qualified or a simple majority? Who has power over agenda‐setting^63^ and who can veto the decisions made? Are there participants who are in some ways dependent on each other or on third parties,^64^, ^65^ such as with an attending physician and a patient?

In already‐established participatory decision‐making settings, organizers can further the process by functioning as a mediator between those involved. Mediation can facilitate the realization of legitimate participation by providing transparency and understanding of the various actors' perspectives. By providing transparency, the represented individuals can understand how decisions are made^66^ and how they will be used, which, in turn, increases public acceptance of the decisions.^67^ By providing a sufficient degree of information and ensuring that appropriate knowledge and understanding of the issue is achieved by the participants, the organizer can facilitate the equal influence of those involved—if desired.^63^, ^67^ By developing and implementing communication strategies to engage stakeholders more effectively,^68^ organizers can ensure that participants' voices are heard and faithfully reflected in the research findings.^66^ Additionally, they can provide representatives with training to enhance relevant knowledge and thereby empower them.^49^ As is often argued, installing moderators to guide discussions can help to mediate between the actors involved by minimizing power differences, by promoting mutual understanding^17^, ^42^, ^67^, ^69^ or by ‘building bridges of trust’ between the actors involved.^70^ ^,^^p.395^ In the context of the FJC, POs are given legal freedom to designate their representatives, but this can certainly not be applied to all conceivable settings in which representation is present. In the event that an organizer selects the participating representatives, the organizer may reflect on relevant responsiveness criteria to reinforce the importance of the setting in terms of decision‐making in advance.

Aside from the abovementioned prerequisites of organizing actors, which are not necessarily present in every conceivable setting, it is often argued that participants, who are representatives themselves, should have additional skills or characteristics that would enhance their ability to appropriately perform the representation of potentially vulnerable individuals.^42^, ^49^, ^59^, ^71^, ^72^, ^73^ At this point, it seems fundamentally useful to distinguish between the two settings—the participatory (representatives and other decision‐makers) and the representative (participants and proclaimed constituency). For the former, skills are needed that enable the representative to assert himself in the decision‐making process. Among the most popular features that a representative should have in this context are communication skills—that is, the representative's ability to express their views, but also to display rational arguments or to negotiate.^42^, ^49^, ^58^, ^67^, ^74^, ^75^, ^76^ Representatives should also be successful operators, who are able to establish strategic alliances with professionals.^42^ They should be independent,^47^, ^67^ should show general interest, determination and motivation,^77^ and should be able to encourage group consensus.^58^ The representatives must also be willing ‘to be persuaded’^78^—that is, they must be willing to change their mind when presented with convincing arguments.^76^ In addition, of course, representatives must have enough time to perform their duties.^50^ In representative settings, on the other hand, skills are needed that enable the representatives to construct the will of their constituency. These certainly include the ability to step into ‘someone else's shoes’, that is, be able to look beyond their own experiences and speak in the name of those being represented.^49^, ^79^ Rather than drawing upon individual feelings, preferences, opinions or health care and research experience, this latter skill implies overcoming or masking one's own feelings to present a case for someone in a potentially very different position.^47^, ^79^

## 5 DISCUSSION AND CONCLUSION

The intention of this article was to sensitize institutions that are planning to conduct or are already conducting participation in decision‐making processes to the importance of representation. We mention three ways in which representation can take place in the health care sector, although these aspects may also be similar in other areas. In the first case, there are mechanisms of authorization and accountability, which together can ensure responsiveness between the representative and the represented. As a second aspect, and closely related to responsiveness, we mention descriptive representation as a possible criterion for potential participants. By trying to involve individuals who are descriptively representative of their proclaimed constituency with regard to their preferences, opinions and needs, such responsiveness might generally be facilitated. As a third aspect, we have mentioned a number of skills that enable representatives to construct the will of their proclaimed constituency on the one hand, and on the other,  to incorporate these contents in the concrete decision‐making process. Taken as a whole, the three criteria mentioned may lead to participation being practiced purposefully in the health care system and elsewhere. Nevertheless, it cannot be emphasized enough that in practice, there can probably be no such thing as ideal‐typical representation, and hence representation will always be imperfect.^79^ This is also due to the fact that the skills and characteristics listed here are utopian in their entirety. No representative will be able to fulfil all of these, and if they do, they are vulnerable anyway if someone intends to question their legitimacy. The listed criteria can therefore rather be described as ideal‐typical. In addition, this paper focused exclusively on how to shape the relationship between representatives and represented, fully acknowledging that this is only one part of a participatory decision‐making process. Arguably, the relationship between representatives and other decision‐makers coming together in a participatory setting is also or primarily essential. In this context, the aspect of power is particularly relevant. Professionals often possess distinctly more power than participating patients and may thus undermine the legitimacy of the latter to maintain control over decision‐making.^64^, ^77^, ^80^ Whenever this becomes apparent in a setting of participation, patient representatives are at risk of becoming mere tokens and thus redundant, and their input is seen as having less priority. Accordingly, in the actual representative setting, power can lead to the positions of those who represent not being heard, which is why, as a consequence, representation might not take place optimally, even if it should be legitimate as such. Further, in the area of descriptive representation, much work is needed—theoretically, methodologically and empirically—to bring the theoretical considerations of the past, which go beyond external characteristics, such as gender or ethnicity, into application‐oriented structures. When exactly a representative qualifies as a descriptive representative —in terms of opinions, preferences and attitudes—remains dependent on a given setting. Similarly, it is highly doubtful that patients, as people with the same condition, share one clear identity that is expressed in the same or even similar opinions and demands.^47^ In terms of representation and participation, it is important to be aware that roles can overlap—professionals can also be patients. It is important to reflect upon the impact of dual roles on patient representation.

Additionally, in the context of this paper, we have not gone into detail about one group of actors, although certainly, this one is of enormous importance for the health sector: self‐authorized representatives. Their representational practices differ substantially from those of formally appointed representatives, but the mechanics of dynamic representation can and should be applied to them as well. Even more, some argue that these actors could be key to establishing comprehensive and pragmatic representation practices.^21^ Research on their concrete contributions to representation in health care is still in its infancy and needs to be expanded. The work that is emerging in this context with POs is, in principle, to be strongly welcomed.

One reason for organizers planning representation insufficiently is that good representation could make them vulnerable and reveal weaknesses that they do not want to be revealed. It may be for this reason that participation is often reduced to a zeitgeist token. Mediating the perspectives of the involved actors in an efficient and appropriate way is a major task and is of importance to science and society as a whole. Due to the relative complexity of representation, representative claims often pass by without being questioned by independent actors. Accordingly, representation is not always a justified means to an end. Quite the contrary, it may increase the danger of the represented people's voices being misused to justify individual acts. Only when legitimate representation is assured, or at least adequately addressed, can this danger be faced in an appropriate way. With the help of legitimate representation, more inclusive and hence fundamentally better decisions can be made. Reflecting on practiced representation may give valuable insight into where violations against legitimacy are visible. In this way, certain forms of representation that are rarely questioned and are thereby stabilized may be overthrown. Instead of taking representation for granted and thus neglecting it in health care settings, we need to elevate the involvement of lay people beyond tokenism by ensuring legitimate representation in all participatory settings. This is particularly relevant for health settings, where some groups are more marginalized than others and seldom heard and where questions of self‐determination and autonomy in the context of vulnerability are always present. Making an appropriate choice of representatives is therefore an ‘ethical necessity’^81^ ^,^^p.246^ (see also Ocloo et al.).^82^ A suboptimal selection of participants, on the other hand, could also mean that ‘those with most to gain are most excluded from health care decision‐making’^45^ ^,^^p.103^ and this could lead to an overrepresentation of the already well‐represented.

One of the main issues with representation is that the complex consequences of poor representation often are not necessarily recognizable as problems of representation, but rather as those of decision‐making arrangements, available resources or for other reasons. If, for example, patients are negatively affected by a decision made in the context of the FJC, in most cases, they may not even know who was present for them in the decision‐making process. Therefore, they cannot make a statement about the representation as such but only judge the decision to be negative for them. In this scenario, representation can hardly be identified as a quality criterion of participatory decision‐making. What follows from this is that one might jump to the hasty conclusion that representation is inherently irrelevant. This may lead to the blanket impression that it is unlikely to make any difference in which individuals are included in any given setting. Nevertheless, the ill‐considered selection of representatives, who neither show a dynamic relationship with the group they claim to represent nor necessarily share their identity or have certain abilities, leads to the risk that these representatives will be unable to give any meaningful input to a given decision‐making process. Furthermore, even though patients and other people represented in such settings might be unwilling to monitor the representative legitimacy of such participatory settings and/or function as a representative themselves,^23^ this should not mean that representation is only considered pro forma. Instead, possible ways to get people involved in interaction with representatives should be explored. This is not just a matter of democratizing the meso‐ and macrolevels of decision‐making in health care; beyond that, an appropriate selection of participants can ensure that the decisions being made are also really in the interests of those who cannot participate themselves. Representation can therefore be seen as a quality feature of participation that can fundamentally ensure the responsiveness of a given health care system, as it may ensure that the needs of the population are met. Thus, representation always sets the course for participation. This most likely is not only true for health care policy‐making but also at low‐threshold levels such as research since even here the selection of ineffective participants can have consequences for the outcome of the respective settings. We, therefore, argue that in most cases where decisions are made with the help of participation, representation is likely to play a role. Even if it turns out that this is not the case, this realization cannot come about without a reflection on representation. This paper may help the actors involved in participatory settings to actively scrutinize their given practices and to change their approaches for the better. At a minimum, this study can help us to carry the discourse on the applicable legitimacy of representation in participatory settings into public debate. Regardless of which direction is pursued, addressing the current status quo is likely to be advantageous.

## CONFLICTS OF INTEREST

The authors declare no conflicts of interest.

## Data Availability

Data sharing is not applicable to this article as no datasets were generated or analysed during the current study.
